# The Response of Patients' Immune System After Facial Mesotherapy

**DOI:** 10.1111/jocd.16792

**Published:** 2025-02-07

**Authors:** Olga Bobowska‐Guglas, Michał Chalcarz, Beniamin Oskar Grabarek, Tomasz Sirek, Jakub Żurawski

**Affiliations:** ^1^ Aesthetic Dermatology Office Olga Bobowska‐Guglas Poznań Poland; ^2^ Chalcarz Clinic Poznań Poland; ^3^ Department of Medical and Health Sciences, Collegium Medicum WSB University Dabrowa Górnicza Poland; ^4^ Department of Plastic Surgery Faculty of Medicine, Academia of Silesia Katowice Poland; ^5^ Department of Plastic and Reconstructive Surgery Hospital for Minimally Invasive and Reconstructive Surgery in Bielsko‐Biała Bielsko‐Biala Poland; ^6^ Department of Immunobiology Poznan University of Medical Sciences Poznań Poland

**Keywords:** immune system, inflammation, mesotherapy

## Abstract

**Background:**

Facial mesotherapy is a minimally invasive cosmetic procedure involving the injection of active substances into the dermis and subcutaneous tissue. Although generally considered safe, complications such as granulomatous inflammation can arise due to improper techniques or unregulated products. This case report highlights the immune response to facial mesotherapy and its clinical management.

**Materials and Methods:**

A 50‐year‐old woman presented with erythematous, hard, and painless facial nodules three weeks after undergoing mesotherapy in a non‐medical setting. A detailed clinical examination, laboratory tests, histopathological analysis, and immunohistochemical evaluation were performed. Tissue expression of CD3, CD20, and CD68 markers was quantified using morphometric analysis. Statistical analysis was conducted to compare the expression of immune markers.

**Results:**

Histopathological examination revealed granulomatous inflammation dominated by macrophages. Immunohistochemical analysis showed significantly higher macrophage activity (CD68, mean reaction area: 5,982.76 µm^2^) compared to T lymphocytes (CD3, 1,775.12 µm^2^) and B lymphocytes (CD20, 187.55 µm^2^) (*p* < 0.001). Initial treatment included antibiotics, corticosteroids, and topical therapies. Subsequent interventions involved intralesional triamcinolone, oral glucocorticoids, and platelet‐rich plasma therapy. Significant clinical improvement was observed within three months, with satisfactory cosmetic outcomes achieved after one year.

**Conclusion:**

Granulomatous inflammation is a potential complication of facial mesotherapy, particularly when performed in non‐medical settings. Effective management requires timely diagnosis, a combination of systemic and topical treatments, and long‐term follow‐up. This case underscores the need for standardized mesotherapy protocols and medical oversight to minimize risks.

## 1 Introduction

Mesotherapy is a minimally invasive procedure used for medical or cosmetic purposes and involves the injection of active substances into the dermis and subcutaneous tissue [1, 2]. Recently, facial needle mesotherapy has become a common treatment with good therapeutic effects. It reduces fine wrinkles and acts as an anti‐aging prophylaxis. Nutritional cocktails used in needle mesotherapy can penetrate deep into the skin, which cosmetics cannot reach.

Although it is a medical procedure, this treatment is currently performed in several beauty salons. Incorrect administration techniques or the use of an unregistered product may result in complications. In addition, it can induce a strong immune response in some cases, resulting in granulomatous inflammation [3].

This case report aimed to perform a semi‐quantitative evaluation of the immunohistochemical expression of cluster of differentiation 3 (CD3), CD20, and CD68 in tissue from a patient who had previously undergone facial microneedle mesotherapy.

## 2 Materials and Methods

### 2.1 Clinical Data

A 50‐year‐old woman visited a dermatologist due to multiple facial skin lesions. According to the information obtained during the medical interview, she had undergone mesotherapy treatment a month earlier at a beauty garden in England, performed by a cosmetologist (non‐medical practitioner) in a beauty salon. We reviewed the patient's history and confirmed that no treatment had been administered in the period before symptom onset and the patient's doctor visit before the doctor's visit. The doctor did not obtain any information regarding the applied product. In addition, the patient did not have any documentation of the procedure she underwent. The first facial skin lesions started to appear approximately 3 weeks after the procedure, and they were red, hard, and painless. They appeared in locations consistent with injection sites as per clinical examination though documentation from the procedure was not available. Four weeks after the procedure, eyelid edema accompanied by itching of the skin, mainly around the eyes, appeared.

**FIGURE 1:**
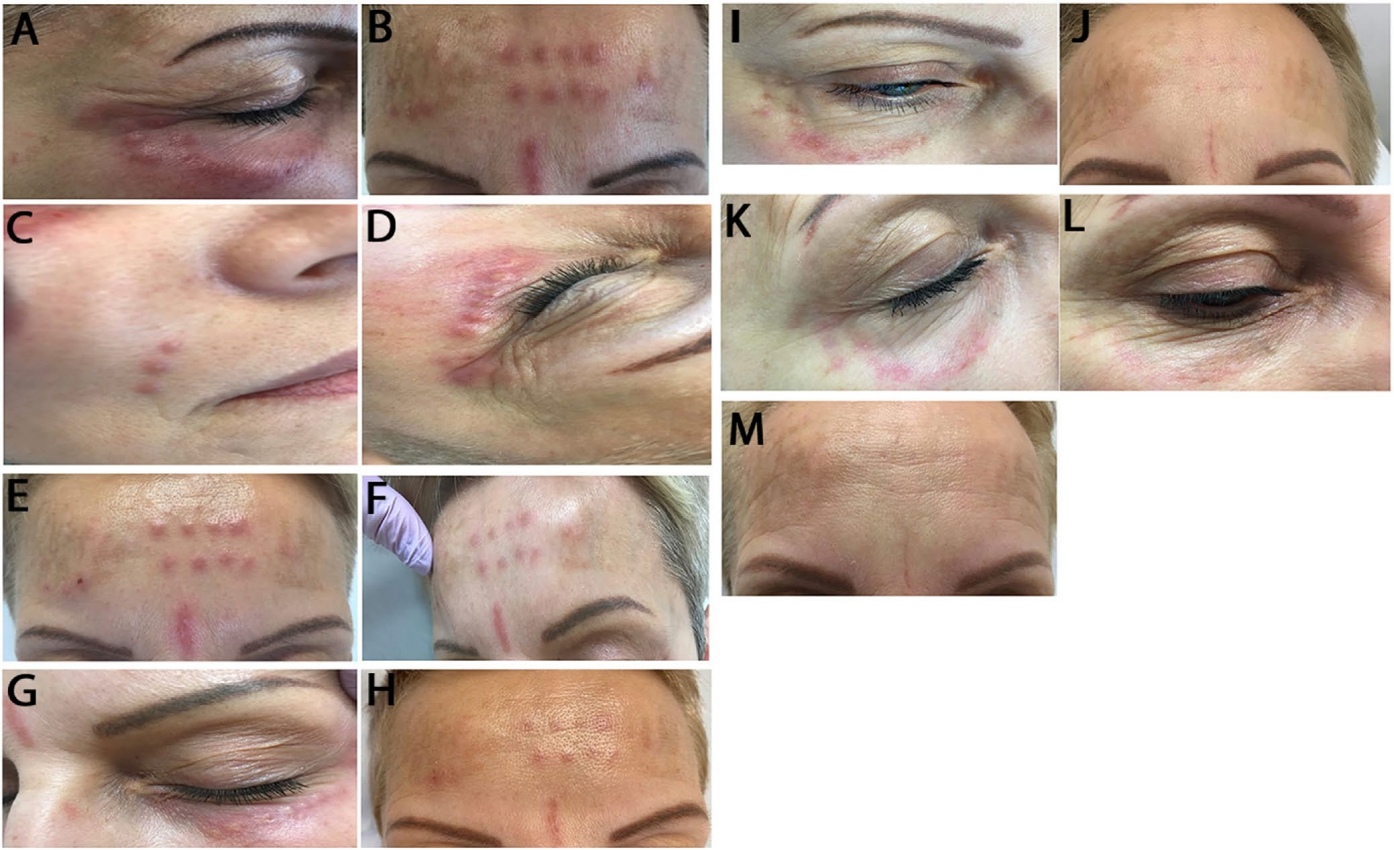
Patient's skin condition before, during, and at the end of treatment. (A) Nodular changes in the orbital area before starting treatment (1st clinical consultation); (B) Nodular changes in the orbital area before starting treatment (1st clinical consultation); (C) Nodular changes in the area of nasolabial folds (1st clinical consultation); (D) Intensification of lesions (2nd clinical consultation); (E) Frontal region (2nd clinical consultation); (F) Reduction of erythema around nodules (3rd clinical consultation); (G) Reduction of erythema around nodules (3rd clinical consultation); (H) 4th clinical consultation; (I) 4th clinical consultation; (J) 5th clinical consultation; (K) 5th clinical consultation; (L) Condition 1 year after the start of treatment—last clinical consultation; (M) Frontal region—last clinical consultation.

During the first clinical consultation (day 31 after the procedure), numerous painless, brown‐red, hard nodules with a diameter of 2–4 mm were observed; these nodules were primarily located around the eyes (Figure 1A), on the forehead and glabella (Figure 1B), and along the nasolabial folds and jawlines (Figure 1C).

Single nodules in the periocular area were painful. The surrounding skin was slightly warmer and swollen. No accompanying adverse effects were observed. The patient did not have any chronic diseases or take any medications on a permanent or temporary basis. Her family history was unremarkable. During the first consultation, a skin biopsy was performed on the lesion in the jawline area; a swab was taken for aerobic and anaerobic bacteria, and laboratory tests were ordered, including morphology, Biernacki test (erythrocyte sedimentation rate), the concentration of C‐reactive protein, glucose, electrolytes, IgE antibodies, anti‐streptolysin O (ASO) test, thyroid‐stimulating hormone, anti‐thyroid peroxidase antibody, anti‐thyroglobulin antibody, and anti‐nuclear antibody pattern 3 profile. The patient's history and clinical presentation indicated granulomatous inflammation and/or biofilm formation.

Based on histopathological findings, which revealed limited inflammatory infiltrates composed mainly of lymphocytes and single epithelial cells suggestive of granulomatous inflammation, the following treatment approach was recommended.

The following treatments were recommended: ciprofloxacin (500 mg 12‐hourly), clarithromycin (500 mg 12‐hourly), probiotics, methylprednisolone (16 mg/day for 2 weeks, followed by a dose reduction to 8 mg/day), bilastine (20 mg/day), potassium supplements, external fusidic acid and hydrocortisone ointments, and fludrocortisone eye ointments.

During the second clinical consultation (65 days after the procedure), the skin lesions became redder, slightly painful, and clearly demarcated (Figure 1D,E).

The patient reported a recent occurrence of throat infection accompanied by fever, which was associated with the worsening of the lesions.

The laboratory results were within the reference ranges, except for the concentration of ASO‐300 IU/mL antibodies (reference value: 10–200 IU/mL). The swab test results were negative.

Triamcinolone (40 mg/mL) was administered intralesionally into the larger nodular lesions, 0.01 mL in each lesion.

Pharmacological treatment was recommended: methylprednisolone (8 mg/day). The antibiotic therapy was changed to amoxicillin/clavulanic acid (1 g 12‐hourly for 14 days, owing to the accompanying upper respiratory tract infections and elevated ASO levels), along with probiotics, potassium supplements, and topical fusidic acid ointment. In addition, control laboratory tests were performed.

At the third consultation (94 days after the procedure), clinical improvement was noted. The injected nodules reduced in size, and the remaining nodules turned pale and softer (Figure 1F). However, reddened nodules around the eyes were still present but were smaller than those observed during previous clinical consultations (Figure 1G).

Laboratory tests revealed that the concentration of ASO antibodies had decreased to 100 IU/mL.

Triamcinolone was injected into the persistent skin lesions at a concentration of 10 mg/mL. The methylprednisolone dose was reduced to 4 mg/day. The oral antibiotic therapy was discontinued.

Boron‐based cream with 10% zinc was recommended for external use.

The fourth consultation was conducted 163 days after the procedure. More than half of the lesions had disappeared (Figure 1H). A single hard nodule with a diameter of 2–3 mm, flesh‐colored, and demarcated around the eyes persisted (Figure 1I).

A mechanical removal procedure was performed on the changes with a plasma generator.

Owing to the coronavirus disease 2019 pandemic, there was a break in treatment. The fifth consultation (276 days after the procedure) took place after 5 months. Single post‐inflammatory hyperpigmentation marks and small atrophic scars were found in the locations of the larger lesions as well as erythematous changes around the periorbital area (Figure 1J,K).

In the next stage of treatment, three series of injections of platelet‐rich plasma were administered at monthly intervals to reduce the visibility of granuloma traces.

After a year of treatment, the patient's clinical condition was assessed, with satisfactory results (Figure 1L,M).

### 2.2 Immunohistochemical Examination

To demonstrate the tested antigens, the following antibodies were used: monoclonal mouse anti‐human CD20cy (Agilent Technologies, Santa Clara, CA 95051, USA; Klon L26, DAKO catalog number M0755), monoclonal mouse anti‐human CD68 (Agilent Technologies, Santa Clara, CA 95051, USA; Klon KP1, DAKO catalog number M0814), and polyclonal rabbit anti‐human CD3 (Agilent Technologies, Santa Clara, CA 95051, USA; DAKO catalog number A0452).

Tissue slides were incubated in a water bath at 96°C in citrate buffer potential of hydrogen 6.0 for 60 min. Endogenous peroxidase activity was blocked using 3% hydrogen peroxide (POL‐AURA, Dywity, Poland). The slides were incubated at 20°C–25°C for 60 min with the appropriate antibodies. The slides were then washed for 10 min in tris‐buffered saline (POL‐AURA, Dywity, Poland). The cells were then incubated with the Dako Real EnVision Detection System Peroxidase/DAB+, Rabbit/Mouse (Dako Cytomation, K5007) for 30 min. In all preparations, DAB‐3,3 chromogen was used to localize the antigen. The slides were then stained with Mayer's hematoxylin, and coverslips were mounted after passing through a series of alcohols to xylene.

For immunohistochemical examination, a reaction in which the primary antibody was omitted was used as the negative control. A tonsillar specimen was used as a positive control.

### 2.3 Cell Imaging With a Light Microscope and Morphometric Analysis

Immunohistochemical studies were performed using an Olympus BX 43 light microscope and an XC 30 digital camera (Olympus, Tokyo, Japan) at a total magnification of 400× (lens magnification 40×).

Quantitative analysis of immunopositive cells was performed based on light microscope images. Calculations were performed using the CellSens Dimension commercial software (Olympus). The program performed a phase analysis of the stained preparation, consisting of the automatic detection of objects based on their color (brown color).

The number of cells and the area of immunohistochemical reactions measured in square micrometers in five fields of view were assessed in the slides. The microscope was calibrated using a calculation program. Threshold values were introduced, according to which the software performed an automatic classification. The results were automatically exported to Excel for further statistical analysis [4].

### 2.4 Statistical Analysis

Statistical analysis was performed using STATISTICA 13PL software (StatSoft, Cracow, Poland). Data for the number of cells and reaction area were presented as mean ± SD, including a 95% confidence interval (CI) for the mean. The normality of the distribution was verified using the Shapiro–Wilk test. Group comparisons were performed using analysis of variance and Tukey's post hoc test. Statistical significance was set at *p* < 0.05.

## 3 Results

### 3.1 Histopathological Examination

**FIGURE 2:**
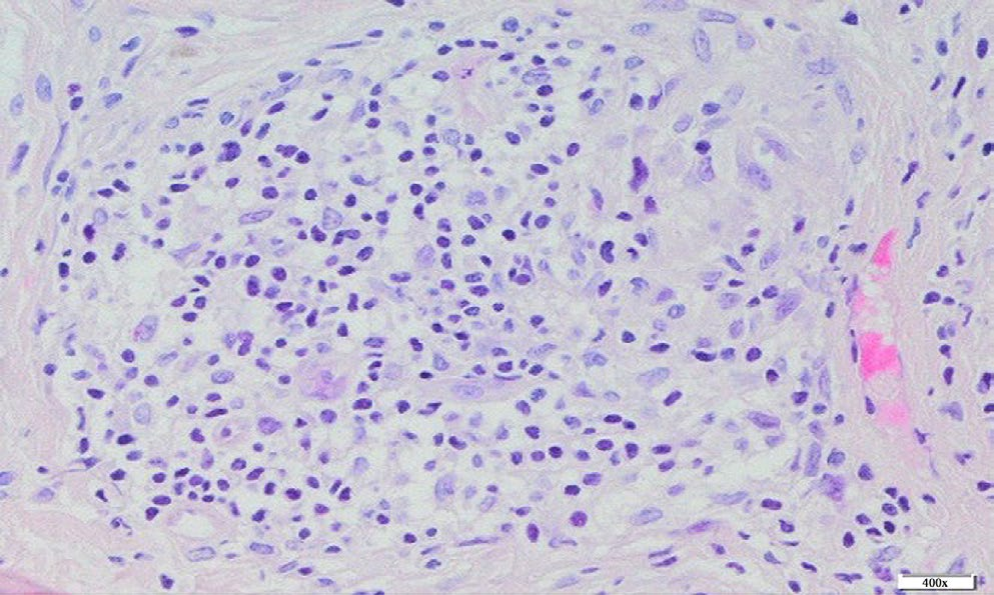
Histological picture of an inflammatory infiltrate consisting mainly of lymphocytes. H&E. 400×.

Histopathological examination revealed small skin fragments with numerous limited inflammatory infiltrates composed mainly of lymphocytes. Single epithelial cells were observed in the examined tissues (Figure 2).

### 3.2 Immunohistochemical Examination

**FIGURE 3:**
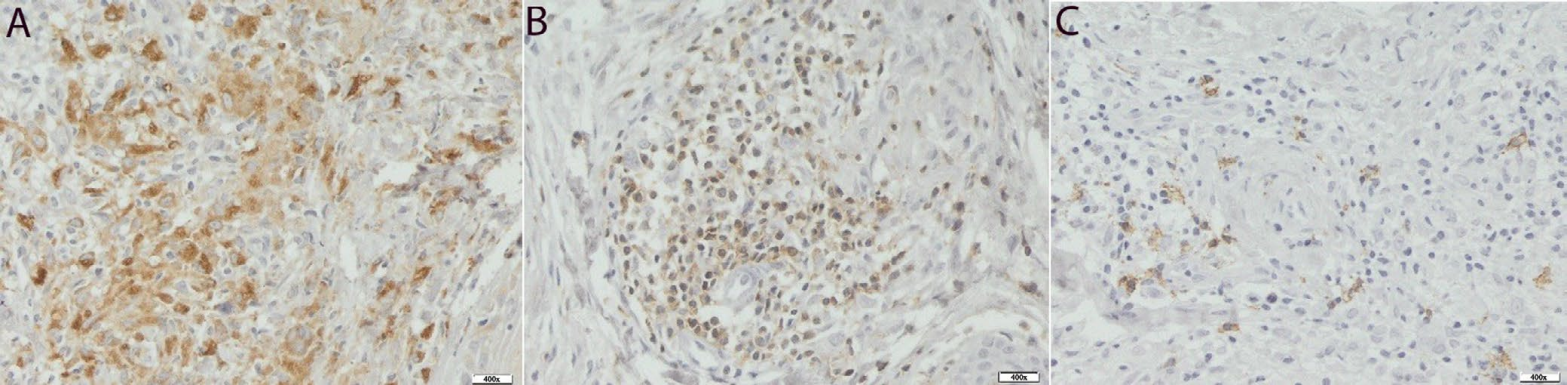
Immunohistochemical expression of: Macrophages (CD 68) (17 A), T lymphocytes (CD 3) (17 B), and B lymphocytes (CD 20) (17 C). Pow 400×.

Tissue expression of all tested markers was observed on histological slides from the patient (Figure 3). The average number of cells was 440.60 (95% CI: 281.49–599.71) for lymphocytes CD3, 42.20 (95% CI: 24.65–59.75) for lymphocytes CD20, and 791.40 (95% CI: 553.38–1029.42) for macrophages CD68. The difference among the three groups was statistically significant (*p* < 0.001), and a post hoc comparison of pairs confirmed that all differences were significant.

The mean reaction area for lymphocytes CD3 was 1775.12 (95% CI: 896.07–2654.16) compared to 187.55 (95% CI: 58.72–316.38) for lymphocytes CD20 and 5982.76 (95% CI: 3927.84–8037.68) for macrophages CD68. The reaction area was significantly different among the three groups (*p* < 0.001). In the post hoc analysis, a difference was confirmed between CD3 lymphocytes and CD68 macrophages (*p* < 0.001) and CD20 lymphocytes and CD68 macrophages (*p* < 0.001); however, the difference between CD20 and CD3 lymphocytes was not significant (*p* = 0.078).

## 4 Discussion

Mesotherapy is considered a relatively safe procedure. Unfortunately, no procedures can be used as standards, primarily in beauty salons. Therefore, several side effects may occur after mesotherapy, the most common being injection site infection [5].

Non‐infectious complications have also been described, primarily foreign body granulomatous reactions after the use of phosphatidylcholine, deoxycholate, buflomedil, silica, carnitine, or various oil‐based substances that are widely used for lipolytic purposes [6, 7]. Another observed side effect is a visible inflammatory reaction resulting in the formation of microabscesses, necrosis of adipose tissue, or the formation of subcutaneous cysts [8].

Granulomatous inflammation is a form of chronic inflammation characterized by the presence of activated macrophages and T lymphocytes. Activated macrophages have granular cytoplasm with distinct boundaries. These cells appear to be epithelial cells. Some of these cells fuse to form multinucleated giant cells. Granuloma formation results from strong T‐lymphocyte activation. The histopathological examination revealed numerous inflammatory infiltrates and single epithelial cells. This type of picture is characteristic of epithelioid granulomas. The mechanism of this inflammation involves macrophages presenting antigens to T lymphocytes after phagocytosis of the causative agent. As a result of this process, normal tissues may be damaged. In granulomatous inflammation, interleukin‐2 is secreted, which is involved in the activation of subsequent T lymphocytes, and interferon‐gamma, which further activates macrophages and changes them into so‐called epithelial cells. Some macrophages can also form giant cells. This condition persists until the antigen or the antigen–antibody immune complex is present [9].

Immunohistochemical expression of all tested markers was observed in the tissue samples examined. Morphometric analysis showed that macrophages play the largest role in the immune response. Half of the T lymphocytes were found compared with macrophages. The lowest expression was observed in the B lymphocytes. The results of our case report allowed us to conclude that the mechanism of granulomatous inflammation described above occurred in this patient. However, the results presented here should be treated as indicative of the small sample size, which is a limitation of this case report.

There are many methods for treating lesions with features of foreign body granulomatous inflammation, depending on the clinical situation, doctor's experience, and type of injected substance. The most frequently preferred therapeutic option is the intralesional injection of triamcinolone at intervals of 4–6 weeks [10, 11, 12] or triamcinolone in combination with 5‐fluorouracil and 1% lidocaine in a ratio of 1:1:1 [13] as well as oral administration of glucocorticosteroids (e.g., prednisone 30–60 mg/day) [11, 12].

Recently, biofilms have been suspected to play a significant role in the formation and progression of foreign body‐type reactions and delayed inflammatory reactions [12]. Additional systemic antibiotic treatment for several weeks is recommended [10, 12, 13, 14, 15], especially a combination of two or three antibiotics, for example, ciprofloxacin 500 mg 12‐hourly [10, 12, 13, 14, 15] and minocycline 100 mg/day or clarithromycin 500 mg 12‐hourly for 3–6 weeks [13].

Other treatment protocols include the administration of empirical antibiotic therapy: clarithromycin 500 mg plus moxifloxacin 400 mg 12‐hourly for 10 days, or ciprofloxacin 500–750 mg 12‐hourly for 2–4 weeks, or minocycline 100 mg/day for 6 months [15]. In cases where the applied substance is confirmed to be hyaluronic acid, intralesional administration of hyaluronidase seems reasonable [11, 12, 13]. Treatment‐resistant or recurrent lesions can be surgically treated [13, 14].

## Author Contributions

Conceptualization, O.B.‐G. and M.C.; methodology, O.B.‐G.; resources, T.S.; data curation, O.B.‐G. and M.C.; writing – original draft preparation, O.B.‐G. and M.C.; writing – review and editing, O.B.‐G., M.C. and B.O.G.; supervision, J.Ż. All authors have read and agreed to the published version of the manuscript.

## Ethics Statement

The study was conducted according to the guidelines of the Helsinki Declaration and was approved by the Bioethics Committee of the Medical University of Karol Marcinkowski in Poznan (protocol code no. KB‐971/23 from 6 December 2023).

## Conflicts of Interest

The authors declare no conflicts of interest.

## Data Availability

The data that support the findings of this study are available from the corresponding author upon reasonable request.
